# A New Supramolecular Tetraruthenated Cobalt (II) Porphyrazine Displaying Outstanding Electrocatalytical Performance in Oxygen Evolution Reaction

**DOI:** 10.3390/molecules27144598

**Published:** 2022-07-19

**Authors:** Hiago N. Silva, Sérgio Hiroshi Toma, Artur Luís Hennemann, Josué M. Gonçalves, Marcelo Nakamura, Koiti Araki, Marcos Makoto Toyama, Henrique Eisi Toma

**Affiliations:** 1 Department of Chemistry, Institute of Chemistry, University of Sao Paulo, Av. Lineu Prestes 748, Butanta, São Paulo 05508-000, SP, Brazil; hiagons@usp.br (H.N.S.); sergioht@gmail.com (S.H.T.); arturhenn@iq.usp.br (A.L.H.); josuemartins@usp.br (J.M.G.); marnak@usp.br (M.N.); koiaraki@iq.usp.br (K.A.); 2 Maua Institute of Technology, Praça Mauá, 1-Mauá, São Caetano do Sul 09580-900, SP, Brazil

**Keywords:** porphyrazine, supramolecular, ruthenium bipyridine, cobalt phthalocyanine, water-splitting, oxygen evolution reaction, electrocatalysis

## Abstract

A new supramolecular electrocatalyst for Oxygen Evolution Reaction (OER) was synthesized from a central multibridging cobalt tetrapyridylporphyrazine (CoTPyPz) species by attaching four [Ru(bpy)_2_Cl]^+^ groups. Both CoTPyPz and the tetraruthenated cobalt porphyrazine species, TRuCoTPyPz, form very homogenous molecular films just by dropcasting their methanol solutions onto GCE electrodes. Such films exhibited low overpotentials for O_2_ evolution, e.g., 560 e 340 mV, respectively, displaying high stability, typically exceeding 15 h. The kinetic parameters obtained from the Tafel plots showed that the peripheral complexes are very important for the electrocatalytic activity. Hyperspectral Raman images taken along the electrochemical process demonstrated that the cobalt center is the primary active catalyst site, but its performance is enhanced by the ruthenium complexes, which act as electron-donating groups, in the supramolecular system.

## 1. Introduction

Current efforts to find alternatives to diminish the emission of toxic and greenhouse gases (such as CO_2_ and CH_4_) in the atmosphere [1] are making fuel cells based on the Oxygen Reduction Reaction (ORR) especially interesting, once the only released product is water. However, this strategy imposes many challenges, such as decreasing the production cost and improving the catalyst efficiency [2]. In fact, water-splitting catalysis is the key factor for the future of such technologies. However, the drawback is the sluggish kinetics involved, especially the anodic oxygen evolution reaction (OER). This leads to high overpotentials [3], impairing the development of commercial routes for oxygen electrochemical production.

**Figure 1:**
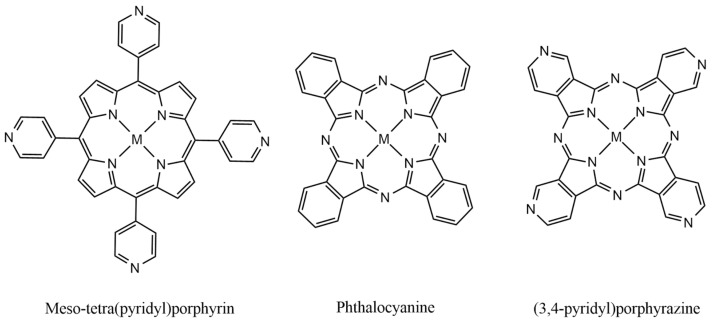
Chemical structures of some MNC metal–organic materials.

Noble metal catalysts such as IrO_2_ and RuO_2_ exhibit a benchmark performance in OER electrocatalysis [4,5], but their scarcity and high cost motivate the search for new alternatives. In this sense, various 3D-metal-based-catalysts, such as oxides, hydroxides and perovskites, have been investigated. Such materials can be promising; however, theoretical predictions hardly match up with experimental results, and precise chemical analysis is also hampered [6]. On the other hand, molecular materials based on Metal–Nitrogen–Carbon (MNC) compounds have been studied as efficient systems for the electrocatalytic synthesis of relevant substrates, such as H_2_, O_2_ and CO_2_ [7]. The “soft materials” encompassing the metalloporphyrins and phthalocyanines are of particular interest [8]. In this group, there is another special system comprising the tetra (3,4-pyridyl) porphyrazines (Figure 1). These species have been little investigated until now, but are very attractive because of their versatile electrochemistry [9], good chemical stability and tunable electronic properties [10]. An outstanding behavior of such compounds is their ability to form molecular films onto electrode surfaces, stabilized by π-π intermolecular interactions and/or electrostatic forces [11].

It should be noted that classical metallo-phthalocyanine (MPc)-based materials, specifically CoPc, have been extensively explored in catalysis [12]. However, a major concern is the stability/durability of their chemically modified electrodes (CMEs). To overcome this problem, polymeric structures, or complex arrangements with metallic linkers and nanocomposites, are frequently employed. Such kinds of materials require fine control in their synthesis to achieve the desired structural properties [13]. In addition, the possible formation of amorphous aggregates can interfere in the study of molecular processes.

Macrocyclic multibridging species exhibit enormous versatility in the construction of organized supramolecular assemblies. In this sense, coordination chemistry can facilitate the design of more complex structures, preserving the properties of the individual groups and promoting cooperative activity from their synergistic interaction [14,15]. In this sense, ruthenium polypyridyl complexes are excellent candidates for the design of functional supermolecules because of their great versatility. Their electrochemical and photochemical features are well established. In particular, the [Ru(bpy)_2_Cl]^+^ species provides suitable Ru^III/II^ redox potential for mediating OER and other oxidation reactions. Combined with the reduction potential of the multibridging center, the possibility of exhibiting bifunctionality increases, making it applicable in catalysis for Hydrogen Evolution Reaction (HER) as well. Furthermore, via the incorporation of positively charged Ru^II^ complexes, it is possible to obtain soluble supramolecular species, in contrast with most porphyrins and phtalocyanines. This facilitates the formation of organized films in different substrates, as well as of electrostatically self-assembled multilayered films. Nanocomposites can also be assembled with negatively charged graphene oxide derivatives.

**Figure 2:**
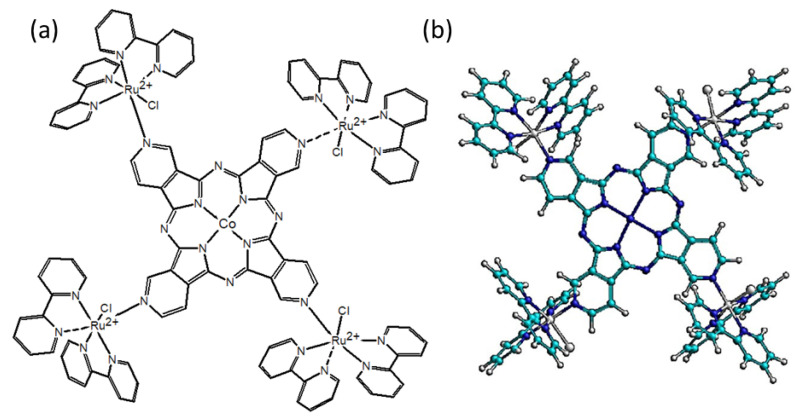
(**a**) Skeletal structure of TRuCoTPyPz and (**b**) ball-and-stick structure of TRuCoTPyPz optimized by ZINDO/S calculations.

Previous investigation in this area has concentrated on the tetraruthenated tetrapyridylporphyrin species [16,17,18,19,20]. Surprisingly, the chemistry of the tetraruthenated tetrapyridylporphrazine analogues remained dormant up to the present time. In this work, a new tetraruthenated cobalt (II) tetrapyridylporphyrazine was synthesized based on the direct assembly of [Ru(bpy)_2_Cl]^+^ groups in each coordinating pyridyl group coupled with the central bridging ligand (Figure 2). This complex revealed an outstanding electrochemical response, particularly in OER catalysis. A detailed electrochemical, spectroelectrochemical and catalytic study of this novel supramolecular species is reported here.

## 2. Results

### 2.1. CoTPyPz Characterization

The CoTPyPz complex is the central unity connecting the peripheral complexes in the supramolecular species reported in this work. Its synthesis was based on the work of Tomoda et al. [21], yielding a dark green solid that is soluble in polar protic solvents, especially in acid conditions. The UV-Vis spectrum in methanol (Figure S1a) shows electronic bands at 207, 285, 340, 415, 580, 636 and 660 nm. The cobalt porphyrazine exhibits typical π→π* bands nominated as M, N or L in the UV region. The broad band at 340 nm and the envelope at 636 and 660 nm are attributed to Soret (B band) and Q (1,0) and Q (0,0) vibronic components, respectively. The 415 nm weak band was assigned to a MLCT transition by UV-Vis spectroelectrochemistry (to be discussed later).

Thermogravimetric analysis was also performed, in addition to elemental analysis, to confirm the purity of the compound and to discard any possibility of contamination with organic byproducts or cobalt precursor complexes. The 10% mass loss under 100 °C (Figure S1b) corresponds to the water content in the samples, in agreement with elemental analysis. The sharp mass loss at 409.5 °C reflects the cobalt oxide formation as the final product of thermodecomposition of the CoTPyPz complex.

### 2.2. [TRuCoTPyPz](TFMS)_4_ Spectroscopy

**Figure 3:**
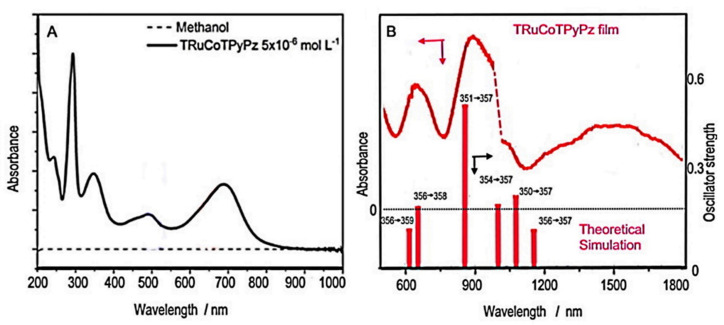
(**A**) Electronic spectrum of TRuCo(II)TPyPz in methanol solution, and (**B**) as molecular film of TRuCo(III)TPyPz onto GCE, including the theoretical bands from ZINDO/S simulation and their corresponding levels. The dotted line in (**B**) refers to instrumental artifact from the changes in the detector module.

The UV-Vis spectrum of the TRuCoTPyPz solution in methanol (Figure 3A) revealed absorption bands with a maximum of 244, 293, 349, 493 and 689 nm. The molar absorptivity for the complex is in the order of 10^5^ mol^−1^ L cm^−1^, preserving the optical characteristics of the isolated molecular units. In a crude approximation, the bands at 293 and 493 nm are characteristic of [Ru(bpy)_2_Cl]^+^ groups attributed to π→π* bipyridine transitions and to Ru(II)→bpy (dπ→pπ*) MLCT, respectively. The bands around 349 and 689 nm correspond to the Soret and Q bands of the porphyrazine ring, respectively.

The electronic spectra of the molecular film deposited on glassy carbon electrode (GCE) were monitored using a specfield instrument, in the visible–near-infrared region, as shown in Figure 3B. Surprisingly, there were strong absorption bands in the near-infrared region, presumably involving Co(III) centers from the air oxidation of the molecular films. This observation indicates that the electronic structure is more complicated than the simple association of the several chromophores, requiring a theoretical approach for its better understanding.

**Figure 4:**
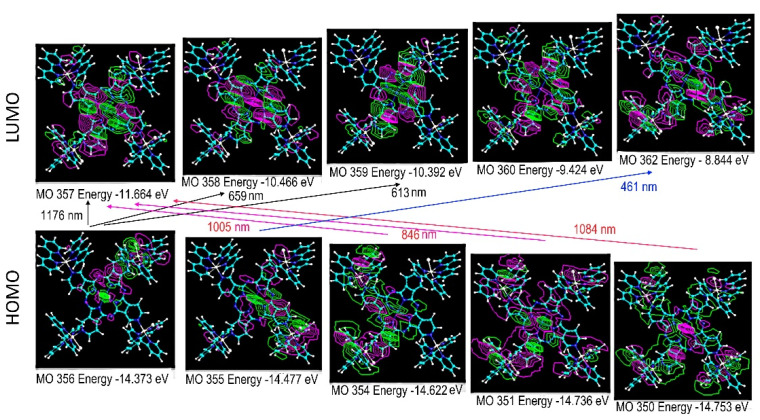
ZINDO/S molecular orbital representations for HOMO and LUMO orbitals of TRuCo(III)TPyPz and the corresponding electronic transitions at 461 (oscillator strength f = 0.116), 613 (0.136), 659 (0.192), 846 (0.588), 1005 (0.375), 1084 (0.215) and 1176 (0.156) nm.

Theoretical TD-DFT calculations of the electronic structure of supramolecular species are usually exceedingly time demanding, and are not feasible using our laboratory computational facilities. For this reason, semi-empirical methods were here employed for convenience. In this regard, it has already been shown that ZINDO/S calculations provide good agreement with the DFT methods [22], and can be applied to more complicated molecules, such as the supramolecular species involved in this work. Therefore, the molecular geometries and electronic levels of the TRuCo(III)TPyPz complexes were calculated using the ZINDO/S program. It should be noticed that the ideal molecular geometry exhibits high symmetry, as shown in Figure 4, displaying an inversion center. This makes the molecule susceptible to Laporte’s parity restriction. 

The ZINDO/S theoretical spectra exhibited six major bands in the visible–near-infrared region at 461, 613, 659, 846, 1005, 1084 and 1176 nm, reproducing qualitatively the observed spectrum in Figure 3. The assignment and illustration of the molecular levels involved can be seen in Figure 3B and Figure 4.

As one can observe in Figure 4, the HOMO levels are strongly delocalized in the supramolecular structure encompassing the Co(III)TPyPz and Ru(bipy)_2_Cl^+^ groups. In contrast, the LUMO levels, except for MO 362, are mainly concentrated on the Co(III)TPyPz center, which behaves as an electron-receptor site. Consequently, all the observed HOMO-LUMO transitions exhibit some charge-transfer characteristics from the peripheral ruthenium groups to the central porphyrazine ring. The fundamental HOMO-LUMO band, corresponding to the MO356-MO357 transition, is theoretically predicted at 1176 nm. However, it is only observed as a shoulder in this region. Presumably, its intensity has been diminished by Laporte´s restriction. The most intense band, at 846 nm, corresponds to the MO351-MO357 transition, exhibiting a strong charge-transfer nature. The second most intense band is expected in the visible region at 659 nm, involving the MO356-MO358 transition, with major participation of the porphyrazine chromophore (Figure 4). This transition corresponds to the observed band at 689 nm (Figure 3A). Another band is theoretically predicted at 461 nm, involving the MO355-MO362 transition (Figure 4). This band encompasses a strong contribution of the Ru(bipy)_2_Cl^+^ moiety to the HOMO and LUMO levels, and corresponds to the observed band at 493 nm in Figure 3A.

FTIR results (Figure S2) show the contribution of the C-C out-of-plane modes and CH and CN stretching modes of the phthalocyanine ring, but the spectrum of TRuCoTPyPz is dominated by the peripheral ligands due to its 4:1 proportion relative to the CoTPyPz center. Bipyridine ring breathing and stretching modes appeared as very strong and well-defined bands in the 500 and 1700 cm^−1^ range [23,24].

**Figure 5:**
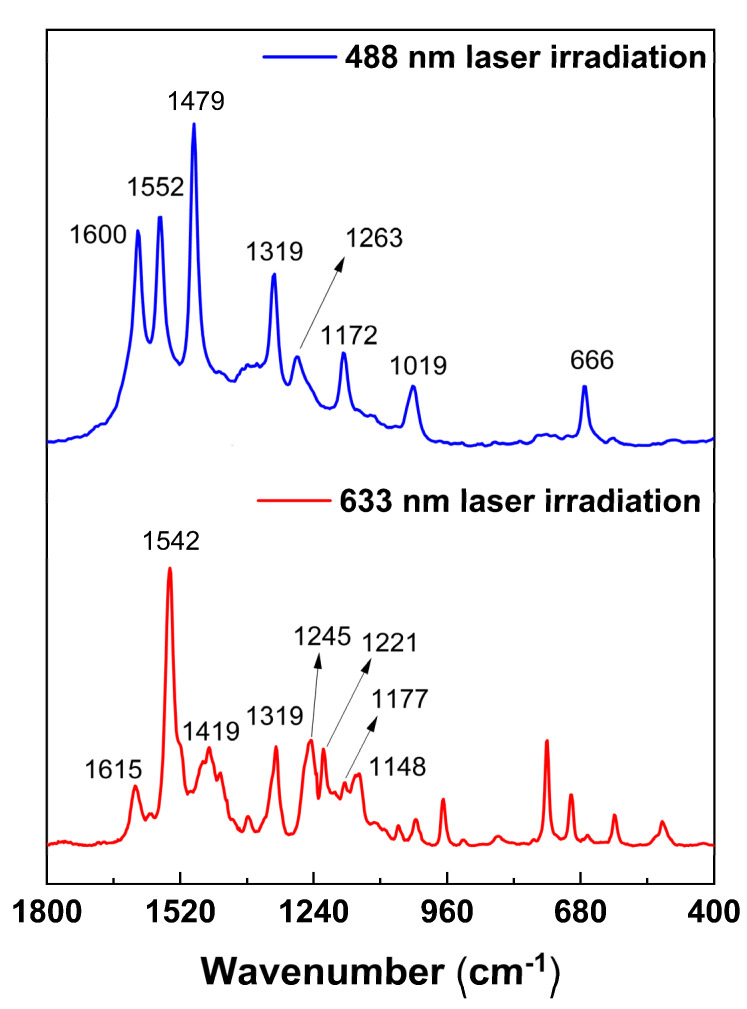
Raman spectra of the supramolecular tetraruthenated complex TRuCoTPyPz. The [Ru(bpy)_2_Cl]^+^ vibrational modes are observed when the sample is irradiated at 488 nm laser (blue line), while the central CoTPyPz vibrational bands are intensified with laser irradiation at 633 nm (red line).

The Raman spectra of the tetraruthenated supramolecular complex revealed distinct profiles when excited at different wavelengths, indicating a resonance Raman effect (Figure 5). The interpretation of Raman data was made in comparison with the literature, by focusing on the spectra of the precursor species (Figure S3).

The Raman spectrum of the TRuCoTPyPz complex obtained at 488nm showed a characteristic pattern of the peripheral ruthenium complexes, in agreement with the assignment of the MO355-MO362 transition at 461 nm (Figure 4). The spectrum is dominated by the bipyridine C−C stretching modes at 1600, 1552, 1479 and 1319 cm^−1^, in addition to the weaker C−H wagging modes at 1263 and 1172 cm^−1^ and Ru−N stretching modes at 1019 and 666 cm^−1^, in agreement with the literature [25]. A small contribution from the porphyrazine ring can be observed as an envelope of weak and broadened bands in the 1700 and 1000 cm^−1^ range. 

On the other hand, by exciting at 633 nm, the Raman spectrum profile closely matched that of the cobalt porphyrazine precursor, indicating resonance with the MO356-MO358 transition at 659 nm (Figure 4). Accordingly, the intense and well-defined peaks at 1615 and 1542 cm^−1^ were attributed to porphyrazine C=C and C=N stretching modes. In addition, there is a series of peaks corresponding to C-N and C-C stretching (1419 and 1319 cm^−1^) and CH bending modes of the pyrrolopyridine groups (1245, 1221, 1177 and 1148 cm^−1^). The bands from 1000 to 450 cm^−1^ can be attributed to the CoTPyPz center as well, including deformation modes of the macrocyclic ring [26,27,28].

### 2.3. Cyclic Voltammetry Behavior

**Figure 6:**
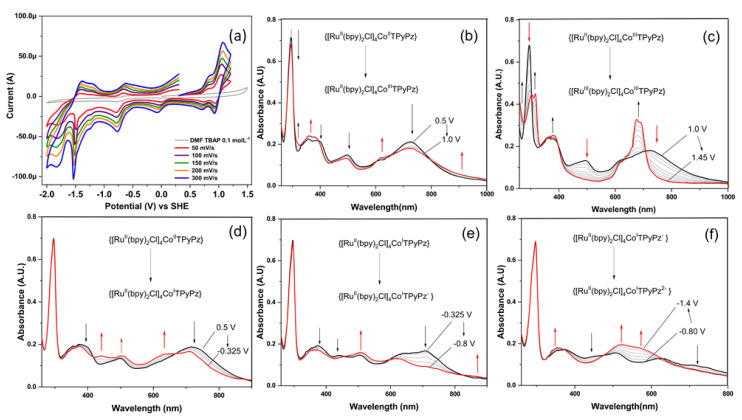
(**a**) Cyclic voltammogram of TRuCoTPyPz followed by (**b**–**f**) its spectroelectrochemistry at different potential ranges: (**b**) from 0.5 to 1.0 V; (**c**) from 1.0 to 1.45 V; (**d**) from 0.5 to −0.325 V; (**e**) from −0.325 to −0.8 V; (**f**) from −0.8 to −1.4 V. Spectroelectrochemical results were recorded shifting the potential in steps of 25 mV.

The cyclic voltammogram of TRuCoTPyPz(TFMS)_4_ in the 1.2 to −2.0 V (vs. SHE) range, using DMF/TBAP 0.1 mol L^−1^ (TBAP—tetrabutylammonium perchlorate) as the electrolyte, exhibits multiple redox processes, as can be seen in Figure 6a. The plots of the currents vs. square root of the scan rates from 50 to 300 mV s^−1^ (Figure S4) indicate linear behavior consistent with diffusion-limited redox processes. The waves at E_1/2_ = 0.79 and 0.97 V can be attributed to the Co^III/II^ and Ru^III/II^ processes, respectively. The 1:4 ratio of the areas under the curves is consistent with the coordination of four [Ru(bpy)_2_Cl]^+^ species to each CoTPyPz unity. In addition, the currents measured for the −0.07, −0.75 and −1.23 V waves are consistent with monoelectronic processes and can be assigned, respectively, to the Co^II/I^ process, and the first and second phthalocyanine reductions to the radical anion and the dianion, in agreement with previously published systems [29,30]. The anodic wave that appears at 0.3 V (Figure S5) is associated with the oxidation of Co^I^ back to Co^II^. All assignments were confirmed by spectroelectrochemical measurements in the potential range of each redox process.

When applying potentials from 0.5 to 1.0 V (Figure 6b), the porphyrazine bands at 400 and 726 nm decay slightly, and new bands appear at 357, 618 and 900 nm, indicating the oxidation of the radical cation species after the oxidation of the Co^II^ ion to Co^III^ coordinated with the porphyrazine ring. The ruthenium complexes also start to oxidize at 1.0 V, explaining the slight changes in the 296, 502 and 464 nm bands associated with the [Ru(bpy)_2_Cl]^+^ moieties, but the main redox process was centered on the cobalt ion at E_1/2_ = 0.79 V. When the applied potential was moved up to 1.45 V (Figure 6c) the changes in the bpy (π→π*) absorption band and disappearance of the MLCT bands at 464 and 498 nm clearly indicate the oxidation of the peripheral Ru complexes to the Ru^III^ state. The intensification of the Q band at 690 nm indicates the increase in the electron-withdrawing nature of the ruthenium complexes upon oxidation of the Ru^II^ sites. 

Turning to negative potentials, the first reduction process was observed at −0.325 V (Figure 6d), decreasing the intensity of the porphyrazine bands at 380 and 724 nm, while leading to the rise of new bands at 442 and 630 nm. These bands are related to a new Co^I^ dπ→Pz(pπ*) MLCT transition and π→π* porphyrazine transition, respectively, in agreement with the reduction of the cobalt ion to Co^I^, as indicated in Figure 6d. In the −0.325 to −0.80 V range, new bands consistent with the radical anion pπ*→pπ* transition rose at 506 and 869 nm after the first reduction of the porphyrazine ring to the radical anion (Figure 6e). Turning to more negative potentials, the anion radical bands decay and bands at 350 and in the 500–600 nm region became stronger at −1.4 V (Figure 6f). These changes are characteristic of the dianion formed by the subsequent reduction of the aromatic CoTPyPz system [31].

### 2.4. Preparation and Characterization of TRuCoTPyPz Thin Films

**Figure 7:**
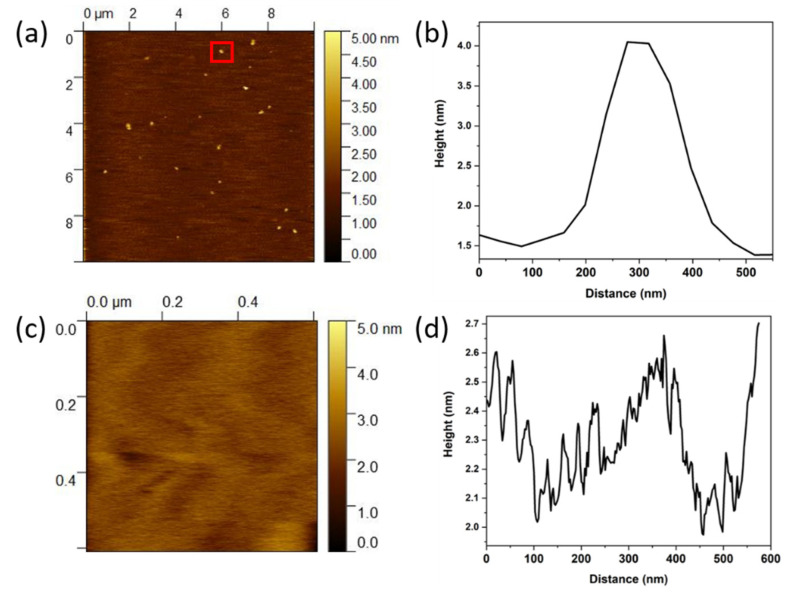
(**a**) AFM of a TRuCoTPyPz thin layer, dropcasted on a mica substrate, showing aggregates as bright spots; (**b**) cross-section of one of the aggregates; (**c**) AFM of the typical TRuCoTPyPz modified GC electrode used as the working electrode in the electrochemical measurements; (**d**) topographic profile of the TRuCoTPyPz electrode.

Thin molecular films of TRuCoTPyPz were firstly characterized by atomic force microscopy by transferring 5 μL of a 1 × 10^−4^ mol L^−1^ methanol solution on the mica surface and letting it dry at room temperature. Figure 7a shows a typical morphology, with numerous columnar structures (Figure 7b) distributed throughout the surface as a consequence of spontaneous aggregation.

The peripheral ruthenium complexes seem to play an important role in the molecular interactions in liquid and solid form. They are responsible for the solubility, as well as for the electrostatic and steric repulsions between the tetracationic supermolecules, keeping them away from each other. During solvent evaporation, they are also responsible for the slow aggregation and spatial organization that results in the morphological features of the aggregates. It has been observed that the Q bands in the 700 nm region remain unchanged, regardless of the concentration. In contrast, the precursor CoTPyPz species promptly forms associated species as the concentration increases [32]. This behavior is also exhibited by previously reported supramolecular porphyrins [11].

The dropcasting technique was utilized to prepare chemically modified electrodes, as follows: 40 μL of the 10^−3^ mol L^−1^ methanol solution of TRuCoTPyPz was dropcast twice onto a GC carbon electrode (GCE) surface, and the solvent allowed to dry in air to form an electroactive molecular thin layer, which covered the entire surface. The AFM image in Figure 7c and the topography in Figure 7d show a uniform molecular film with RMS roughness < 0.5 nm. These features observed for TRuCoTPyPz film on the electrode surface contribute to the system’s higher electrocatalytic activity.

### 2.5. Electrocatalytic OER

**Figure 8:**
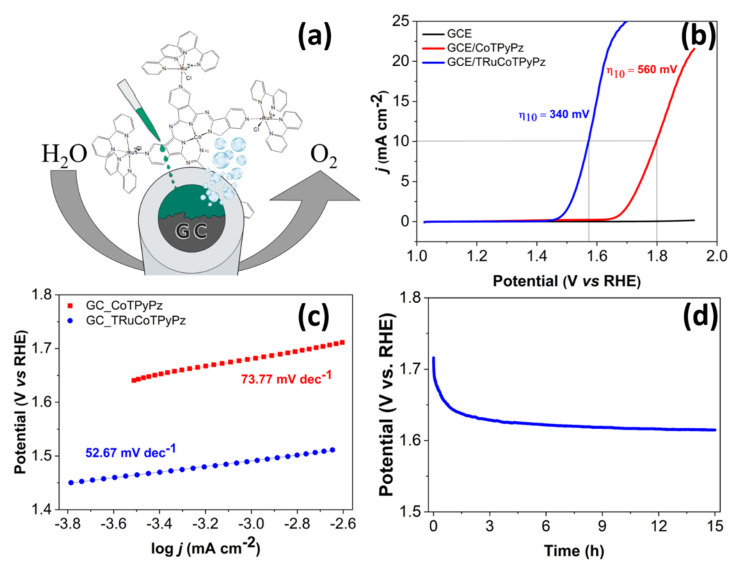
(**a**) General scheme of the molecular film onto GCE for O_2_ evolution. (**b**) LSV polarization curves at v = 5 mV s^−1^ in 1.0 mol L^−1^ KOH solution and corresponding (same color) Tafel plots (**c**) for CoTPyPz/GCE and TRuCoTPyPz/GCE. (**d**) Potential as a function of time registered during electrolysis (OER) using GC electrodes modified with TRuCoTPyPz.

The OER performance of TRuCoTPyPz was evaluated in alkaline medium at room temperature and compared with that of the CoTPyPz precursor (Figure 8). Accordingly, the surface of rotating disk glassy-carbon electrodes (GCE) was modified separately, with CoTPyPz and TRuCoTPyPz, for further evaluation of the electrocatalytic activity of the immobilized materials. The CoTPyPz/GCE- and TRuCoTPyPz/GCE-modified electrodes were assembled in a conventional three-electrode electrochemical cell, and the polarization curves were recorded in 1.0 mol L^−1^ KOH electrolyte solution using linear sweep voltammetry (LSV), at a scan rate of 5 mV s^−1^. Voltammograms were recorded from 1.0 to 3.0 V vs. RHE at pH 14, and the respective Tafel plots evaluated, as shown in Figure 8b or Figure 8c. A rotation rate of 1200 rpm was employed to assure efficient removal of eventually formed dioxygen bubbles. Interestingly, the overpotential value at 10 mA cm^−2^ decreased continuously (Figure S6), indicating an improved electrochemical performance of the electrode material as a function of operation time, apparently involving some catalyst activation [33]. This reflects on the decrease in the Tafel slope values as a function of the number of scans, confirming the improvement of the heterogeneous reaction kinetics. However, this behavior was much more pronounced in the first five scans, after which the electrocatalytic performance of the modified electrode remained essentially constant.

A good electrocatalyst for OER must support a high current density at the smallest overpotential as possible, e.g., at a current density of 10 mA cm^−2^ or higher [34]. In fact, the results obtained with TRuCoTPyPz/GCE were significantly much better than that for the CoTPyPz/GCE, as indicated by the lower overpotential (η_10_) of 340 mV (TRuCoTPyPz/GCE) compared to 560 mV for the CoTPyPz/GCE (Figure 8b). In addition, the plot of potential against log *j* [35], known as the Tafel slope, is also an important parameter related to the reaction kinetics of electrocatalysis (Figure 8c). The TRuCoTPyPz electrocatalyst showed a Tafel slope of 52.67 mV dec^−1^, which was much lower than the 73.77 mV dec^−1^ determined for CoTPyPz. These overpotentials and Tafel slope values are similar to those reported for other molecular electrocatalysts and noble metal oxides (Table 1), indicating that TRuCoTPyPz electrocatalyst is promising for OER. In fact, as can be seen in Figure S7, the TRuCoTPyPz/GCE has a lower Rct than CoTPyPz/GCE in the electrochemical impedance spectroscopy (EIS) measurements, which is consistent with its higher electrocatalytic activity. Finally, TRuCoTPyPz was stable for up to 15 h at operation conditions simulated by chronopotentiometry (Figure 8d), at a current density of 10 mA cm^−2^. The film showed a stabilization pattern during the first 3 h, which is coherent with the catalyst activation step. After this, the film response remained practically constant for at least 15 h. It remained strongly adhered to the surface as a dark smooth film, while preserving its electrochemical activity.

The difference in the electrochemical behavior of the precursor cobalt porphyrazine and the TRuCoTPyPz is evident, indicating that the coordination of Ru complexes in the macrocycle ring periphery contributes to the boost in its catalytic activity. The first and major concern was regarding the role of [Ru(bpy)_2_Cl]^+^ groups in the supramolecular material, as to whether they would act as isolate catalytic centers or synergetically enhance the central CoTPyPz catalytic properties.

The voltammogram in aqueous medium at neutral pH showed the reversible wave at 0.8 V vs. SHE, which was characteristic of the coordinated [Ru(bpy)_2_Cl]^+^ groups [Ru^III^/Ru^II^] couple, and not of high-valence species such as [Ru^IV^=O]^2+^/[Ru^V^=O]^3+^, which is generally found at 1.6–1.7 V vs. SHE [45]. Interestingly, the reduction wave associated with the ruthenium complexes was no longer observed after the OER, indicating that the starting Ru^II^ species was oxidized during the electrocatalytic process (Figure S8). The formation of Ru^IV^=O was improbable because the chloride ligand remained coordinated and was not substituted by water [46]. In addition, the wave assigned to the first reduction of the porphyrazine ring remained unchanged, confirming the integrity of its structure. The peripheral [Ru(bpy)_2_Cl]^+^ groups probably enhanced the stability of the porphyrazine core via electronic effect, since they are oxidized at lower potentials, starting to act as electron-withdrawing substituents. This behavior results from the significant electronic coupling between their parts, as confirmed by UV-Vis spectroelectrochemistry and theoretical simulation, which indicate a charge-transfer effect from ruthenium complexes to cobalt porphyrazine moieties.

The chemical integrity of the film after performing 15 h of chronopotentiometry was monitored by Raman spectroscopy, as shown in Figure S9. The Raman pattern of the films was essentially preserved, although there was a small enhancement of the peaks coinciding with the GCE disk signals, indicating a minor release of material after this time.

In fact, TRuCoTPyPz possessed the lowest charge-transfer resistance (Rct) of 3.85 Ω, comparable to that of polymeric cobalt phthalocyanines nanomaterials [10], as measured by electrochemical impedance spectroscopy, EIS (Figure S7). The synergy between those metallic centers and the contribution of the periphery ligands in the electronic conduction mechanism probably enhanced the electrochemical properties of the supermolecular material. That hypothesis is in agreement with the results previously found for free-base tetraruthenated porphyrazine [11], which demonstrated that the electrocatalytic properties of TRuCoTPyPz can be tuned by the ruthenium complexes coordinated to the periphery of the macrocyclic compound.

The mechanism of OER mediated by metallophthalocyanines in alkaline conditions involves a metal oxyhydroxide species intermediate as the rate-determining step, in one of the four steps of the multielectron reaction [47] shown in Equations (1)–(4). To confirm the formation of the peroxo-adduct, confocal Raman imaging was conducted, since the band at 565 cm^−1^ attributed to a Co-(O-O)^2−^ bond [48], which was initially absent, appeared in the oxidized electrode material. For this purpose, the sample was prepared by modifying fluorine-doped tin oxide (FTO) glass electrodes with TRuCoTPyPz and chemically mapping via Raman microscopy using WITEC confocal Raman equipment and a piezo-driven sample holder.

M + OH^−^ → MOH + e^−^ (1)

MOH + OH^−^ → MO* + H_2_O + e^−^ (2)

MO* + OH^−^ → MOOH + e^−^ (3)

MOOH + OH^−^ → M + O_2(g)_ + H_2_O_(l)_ + e^−^ (4)

**Figure 9:**
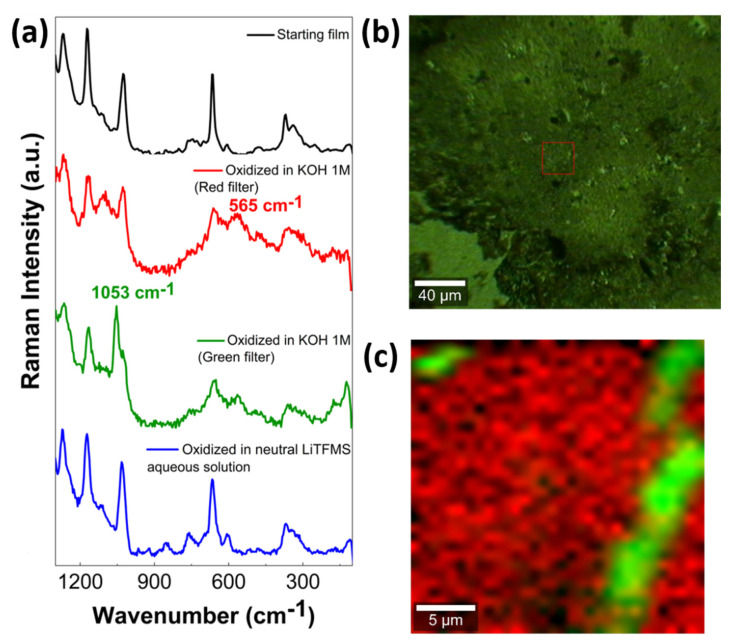
(**a**) Raman spectra of TRuCoTPyPz films on FTO electrodes before (black line) and after electrocatalytic oxygen evolution reaction in 1 M KOH in the red and green filters of the Raman mapping. The blue line represents the Raman spectra of the TRuCoTPyPz film oxidized in neutral lithium triflate aqueous solution in the absence of oxygen; (**b**) optical microscopy image of the TRuCoTPyPz film on FTO glass substrate; (**c**) Raman spectral mapping of the marked region in (**b**).

The Raman microscopy of the TRuCoTPyPz electrode was carried out via probing with a 488 nm laser. No significant difference was found before and after oxidation without oxygen saturation. However, a band typical of an M-O complex was found at 565 cm^−1^ in the sample electrochemically oxidized in 1 M KOH solution. Furthermore, an additional band that could be attributed to a O-O stretching mode was found at 1053 cm^−1^ in the same sample, indicating the formation of the intermediate Co-OOH (Figure 9a). No such similar bands typically assigned to a Ru-O vibrational mode and/or Ru-OOH species were observed [49].

An optical image of a typical TRuCoTPyPz-modified FTO electrode (Figure 9b) revealed some irregularities on the surface, which were different from those found on thicker films on GCE used in electrocatalysis. The brightest parts are related to valleys and holes where the metal–oxygen species are concentrated, as represented by the red contrast. The Raman mapping monitoring the band at 565 cm^−1^ (red filter) and at 1053 cm^−1^ (green filter) was superimposed to show the spatial superposition of the sites concentrating the characteristic M-O stretching and O-O stretching modes (Figure 9c). However, the activity is related to the flux of charge carriers, which becomes larger as the film becomes thinner and the electric conductivity increases. Other regions of the electrode surface reproduce the same profile. This result indicates that intermediate oxyhydroxide species are formed in higher yields when the catalysis is closer to the electrode surface, allowing a faster electron transfer. In short, the OER process proceeds via the oxidation of the hydroxo ligand coordinated at the CoTPyPz site, forming a peroxide species, which is converted into dioxygen and finally released.

## 3. Materials and Methods

### 3.1. Materials and Instruments

All reagents and solvents were purchased from Sigma Aldrich and used without further purification. DMF, n-butyl alcohol, chloroform, acetic acid, methanol, ethanol, 2,2,2-trifluoroethanol and other solvents were used as received from the manufacturer with purity > 99%.

UV-Vis absorption measurements of solution were taken in an Agilent HP8453 UV-Vis spectrophotometer in a standard quartz cuvette with a path length of 1 cm. Absorption measurements of the films were obtained using a Vis-NIR spectroradiometer ASD Inc. FieldSpec3.

FTIR spectra were made with a Bruker Alpha IR spectrophotometer with a transmittance module, and the samples were prepared in KBR pallets.

Electrochemistry measurements were conducted in a Metrohm Autolab pgstat30 potentiostat using a three-electrode cell, glassy-carbon electrode (GCE) as the working electrode, with a geometric area of 0.2475 cm^2^; a platinum mesh as the counter electrode; and Ag/AgCl in aqueous KCl 3M as the reference electrode. To obtain the Tafel slope of Tetraruthenated cobalt porphyrazine, Tafel plots were acquired from LSV polarization curves using the Tafel equation (Equation (5))

*η* = *b* log *j* + *a* (5)

where *η* (expressed in mV), *j* (in mA cm^−2^) and *b* (in mV dec^−1^) are the overpotential, current density and Tafel slope, respectively.

The AFM images have been obtained using a FlexAFM (NanoSurf, Liestal, Switzerland) microscope attached to C3000 controller (Nanosurf) operating at Intermittent Contact AFM Mode with TAP 190 Al g cantilevers (Budget Sensors, Sofia, Bulgaria). To observe the TRuCoTPyPz molecular aggregates, the AFM sample was prepared by dropcasting 5 μL of TRuCoTPyPz diluted solution on mica surface (Ted Pella Inc., Redding, CA, United States) previously cleaved. 

Confocal Raman microscopy measurements were performed with WITEC alpha 300R equipment using an excitation laser at 488 nm, 532 nm and 633 nm. Spectral images were obtained by scanning the sample in the x, y direction with a piezo-driven xyz feed-back controlled scan stage and collecting a spectrum at every pixel. All experiments were conducted with a Nikon objective (20 × NA = 0.9), applying a laser power lower than 18 mW/cm^2^ on the sample stage. Raman images were generated from the band height intensities measured at the maximum of the peaks.

Semiempirical ZINDO/S calculations [22] were carried out using the Hyperchem 8.05 computational package, combining interactive MM^+^ geometry and CI calculations, by performing optimization cycles up to a convergence limit of about 10^−5^ kcal Å^−1^ mol^−1^. The electronic distribution was generated from single CI excitations in an active space involving 20 frontier molecular orbitals (10 highest occupied and 10 lowest unoccupied MOs).

### 3.2. Synthesis of Precursors Ru(bpy)_2_Cl_2_ and CoTPyPz

Complex Ru(bpy)_2_Cl_2_ used as peripheral ligand was synthesized, reacting RuCl_3_.3H_2_O, 2-2′-bipyridyne and LiCl in excess (1:2:10). The mixture was refluxed in DMF, as described previously [50]. Elemental Analysis for C_20_H_18_Cl_2_N_4_ORu exp (calc.): C 48.16% (47.82%); H 3.25% (3.61%); N 11.20% (11.15%).

For the synthesis of the bridging central unit, 405.5 mg of 3,4-dicyanopyridine and 204.8 mg of cobalt (II) acetate were dissolved in 60 mL of n-butyl alcohol with 1,8-diazabicyclo [5.4.0]undec-7-ene (DBU) as catalyst. The reaction mixture was refluxed for 24 h. After it was cooled to room temperature, the viscous product was diluted with concentrated chloride acid and CHCl_3_ was added to remove organic impurities. The mixture was separated in a separatory funnel, collecting the porphyrazine aqueous phase. The metal porphyrazine was precipitated when NaOH saturated solution was added, until pH 9. The fine powder was filtered and washed exhaustively with water and ethanol. After drying at 80 °C, the compound was again suspended in diluted acetic acid as a purification method. This step was repeated until the filtered substance remained colorless. The solid was once again dried in the oven, resulting in a dark powder with the estimated yield of 60%. Elemental analysis for C_30_H_32_CoN_12_O_10_ (0.3 C_2_H_6_O) exp. (calc.): C 46.18% (45.88%); H 4.17% (4.46%); N 19.44% (19.69%).

### 3.3. Synthesis of TRuCoTPyPz

Following the method previously described, 182 mg of [Ru(bpy)_2_Cl_2_].H_2_O (0.37 mmol) and 49.6 mg of CoTPyPz (0.086 mmol) were added in 2,2,2-trifluoroethanol and refluxed for 1.5 h. The solvent was completely evaporated under vacuum and went back to reflux in methanol for 3 h to guarantee the coordination of the four [Ru(bpy)_2_Cl]^+^ groups. The solvent was again removed, the resultant solid was dissolved in minimum DMF and the compound was readily precipitated in lithium triflate aqueous solution. After filtration, the solid was washed copiously with water and ether and dried under vacuum. Finally, the compound was purified in column chromatography in neutral alumina, using DCM/methanol as eluent. The precipitation step was repeated, and 146 mg of the complex was obtained with 54.44% yield. Elemental analysis was conducted for C_112_H_76_Cl_4_CoF_12_N_28_O_12_Ru_4_S_4_.7H_2_O Exp. (Calc.): C 43.55(43.49); H 3.07 (2.93); N 12.60 (12.68).

## 4. Conclusions

The electrochemistry of the porphyrins and phtalocyanines can be further improved by generating supramolecular species, encompassing additional metal complexes in the structure. Such complexes can help stabilize the system, increasing the solubility, improving the reactivity and facilitating the formation of molecular thin films, especially for optical and electrochemical exploration. In this work, such a strategy was successfully applied to the cobalt tetrapyridylporphyrazine system (CoTPyPz). Accordingly, a new supramolecular species was elaborated by attaching four [Ru(bpy)_2_Cl]^+^ groups in the peripheral pyridyl groups of CoTPyPz. Spectroscopic and electrochemical evidence, supported by theoretical calculations, indicated that the electron acceptor characteristics of the cobalt tetrapyridylporphyrazine center perfectly match the electron donor properties of the ruthenium(II) moieties. This complementary aspect gives rise to synergistic effects, improving the electrochemical behavior of the supramolecular species. In this way, a highly active film of TRuCoTPyPz was formed by dropcasting onto GC electrode, showing a good response for the OER process. Notably, there was an enhancement in the electrochemical performance of the TRuCoTPyPz film in comparison with the precursor CoTPyPz, as observed from the LSV measurements in the current density of 10 mA cm^−2^ in 1 M KOH solution, with an overpotential of 340 mV and Tafel slope of 52.67 mV dec^−1^, respectively. A synergistic mechanism is implied, involving the conjugated action of Co and Ru in the bimetallic catalyst, generating a Co-OOH species, as observed in the Raman hyperspectral images. The novel supramolecular species exhibited better electrocatalytic performance in OER processes in comparison with the similar molecular systems, which was practically comparable with the commercial RuO_2_ and IrO_2_ electrocatalysts.

## Figures and Tables

**Table 1:** Comparison of the electrochemical parameters of some OER electrocatalysts.

Catalyst	η_10_ (mV)	Tafel Slope (mV dec^−1^)	Electrolyte	Ref.
RuO_2_	309	62	1 M KOH	[34]
IrO_2_	320	76	1 M KOH	[36]
1:1 polyTACoPc + IrO_2_	304	39	0.1 M KOH	[37]
FeNi-COP-800	400	103	0.1 M KOH	[38]
Fe_0.5_Ni_0.5_Pc-CP	317	116	1 M KOH	[39]
CoTCPP/ZrP	476	76.4	0.1 M KOH	[40]
CoTMPP@ZIF-8	387	210.3	0.1 M KCl	[41]
CoP-2ph-CMP-800	370	86	1.0 M KOH	[42]
Co-MPPy-1	420	58	1.0 M NaOH	[43]
PCOF-1-Co	386	89	1.0 M KOH	[44]
CoTPyPz	560	74	1 M KOH	This work
TRuCoTPyPz	340	53	1 M KOH	This work

## Data Availability

All the raw data of this research can be obtained from the corresponding authors upon reasonable request.

## Supplementary Materials

The following supporting information can be downloaded at https://www.mdpi.com/article/10.3390/molecules27144598/s1, Figure S1. (a) UV/Vis absorption spectra and (b) TGA curves of CoTPyPz. Figure S2. FTIR spectra of the TRuCoTPyPz powder on KBr pallet. Figure S3. Raman spectra of the isolated precursors ruthenium bipyridinium complex (red), cobalt tetrapyridyl porphyrazine (blue) and the supramolecular tetraruthenated complex TRuCoTPyPz (black) obtained at 488 (A), 532 (B) and 633 nm (C) lasers irradiations. Figure S4. Plots of the current vs. the square root of the scan rates for the ruthenium oxidation wave at 0.97 V (black line), cobalt oxidation process at 0.79 V (red line), first reduction of TPyPz center at −0.75 V (blue line), second reduction of TPyPz at −1.23 V (green line) and bpy reduction at −1.5 V. Figure S5. Cyclic voltammetry of TruCoTPyPz in DMF containing TEAClO_4_ 0.1 M at several scan rates. Figure S6. Successive LSV polarization curves at v = 5 mV s^−1^, in 1.0 mol L^−1^ KOH solution (A) and corresponding (same color) Tafel plots (B) for TRuCoTPyPz. The working electrode was constantly rotated at 1200 rpm to remove the O_2_ bubbles (for interpretation of the references to color in this figure legend, the reader is referred to the Web version of this article). Figure S7. EIS data for GC electrodes modified with CoTPyPz and TRuCoTPyPz. Nyquist plots at DC potential of 1.573 V vs. RHE. The EIS measurements were recorded superimposing an AC perturbation (ΔE = 10 mV) in the 0.01 to 10,000 Hz range, after five successive LSVs in the 1.025–1.625 V vs. RHE range, at a scan rate of 5 mV s^−1^. Figure S8. Cyclic voltammetry of TRuCoTPyPz film on glassy carbon electrode in neutral aqueous LiTFMS 0.1M at 50 mV s^−1^ showing redox processes before and after chronopotentiometry of 15 h. Figure S9. Raman spectroscopy of the TRuCoTPyPz film obtained with 488 nm laser wavelength before and after 15 h chronopotentiometry. In green, the initial TRuCoTPyPz film in GCE rotating disk; in blue, spectrum obtained in the middle of the GCE disk; in orange, measurements obtained at the periphery area of the GCE disk; and in black, spectrum of the bare GCE disk electrode.

## Sample Availability

Not available.
