# Supplementary material for: A New Supramolecular Tetraruthenated Cobalt (II) Porphyrazine Displaying Outstanding Electrocatalytical Performance in Oxygen Evolution Reaction
Source: Molecules. 2022 Jul 19;27(14):4598. doi: 10.3390/molecules27144598 (PMC9318768; doi:10.3390/molecules27144598)
Supplement: Supplementary file 1 [file molecules-27-04598-s001.zip › molecules-1817313-supplementary.pdf]

Supplementary Files

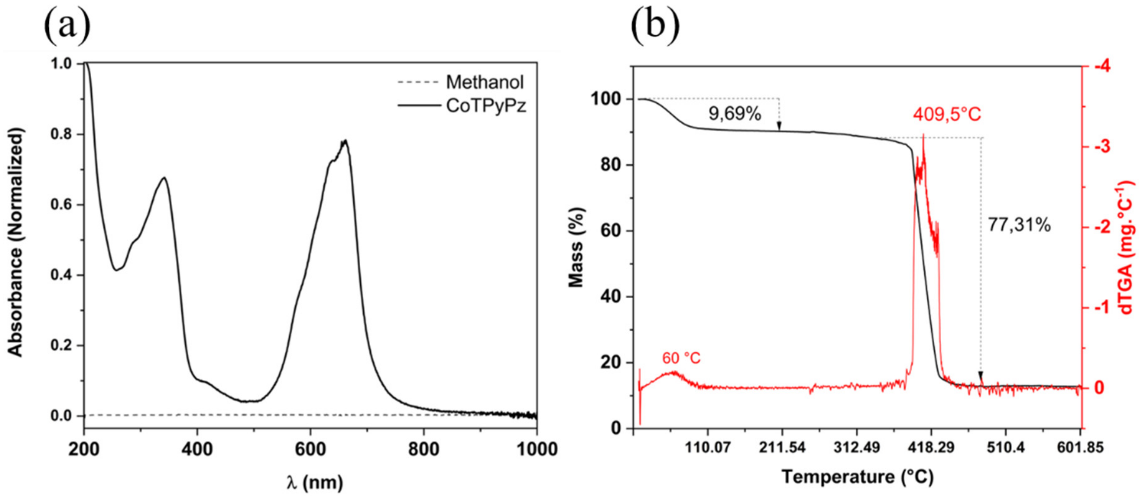

Figure S1. (a) UV/Vis absorption spectra and (b) TGA curves of CoTPyPz.

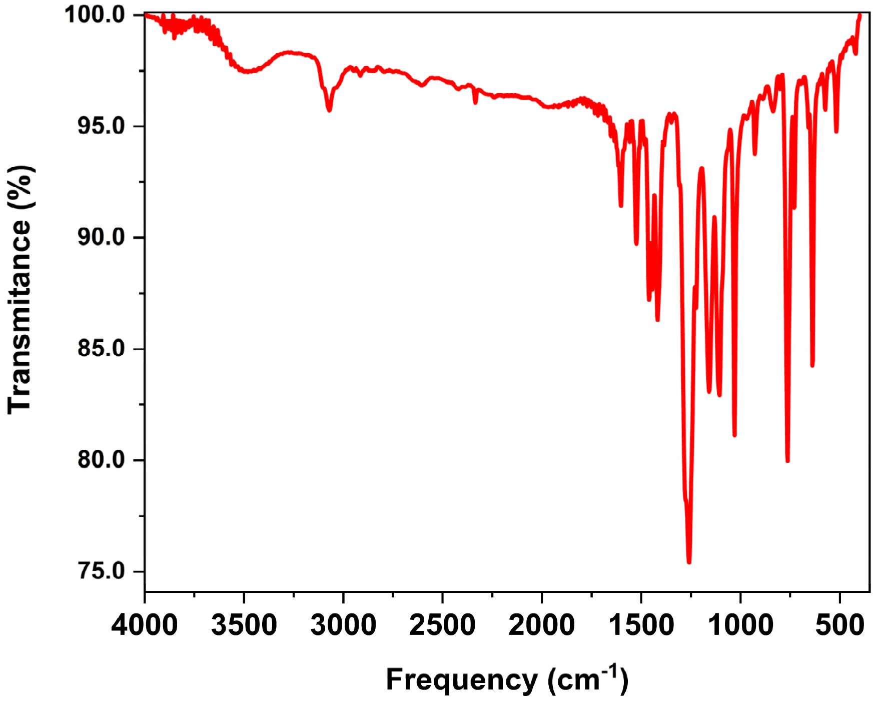

Figure S2. FTIR spectra of the TRuCoTPyPz powder on KBr pallet.

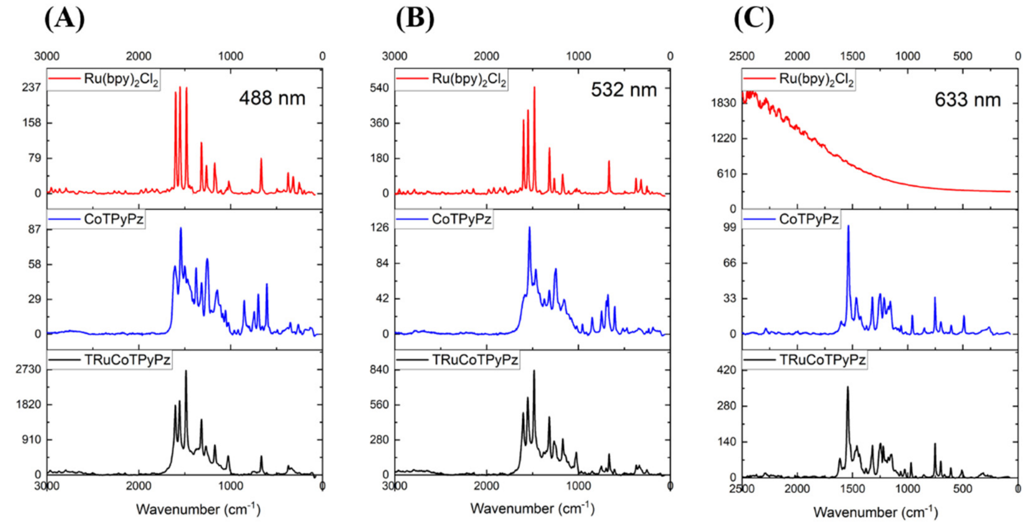

Figure S3. Raman spectra of the isolated precursors ruthenium bipyridinium complex (red), cobalt tetrapyridyl porphyrazine (blue) and the supramolecular tetraruthenated complex TRuCoTPyPz (black) obtained at 488 (A), 532 (B) and 633nm (C) lasers irradiations. Interestingly, at 633 nm, the Raman spectrum of the ruthenium bipyridinium complex showed only a broad band pattern, with no defined peaks, which can be attributed to an autofluorescence process of the complex.

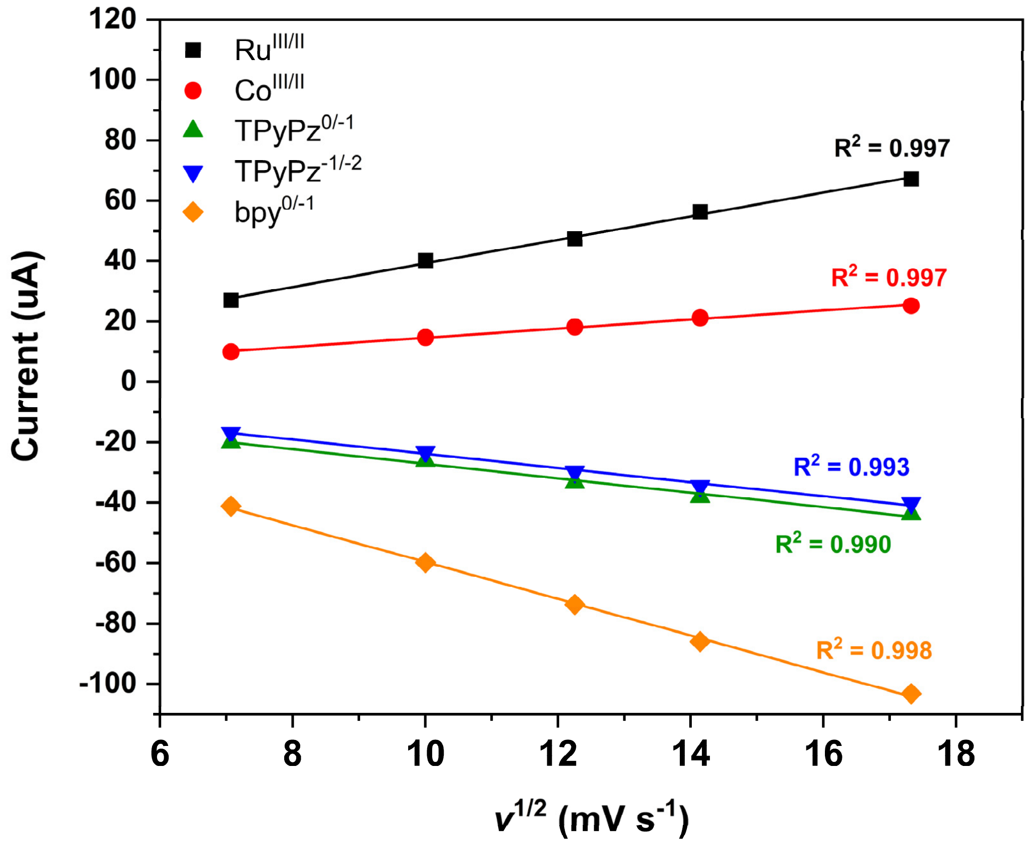

Figure S4. Plots of the current *vs.* the square root of the scan rates for the ruthenium oxidation wave at 0.97 V (black line), cobalt oxidation process at 0.79 V (red line), first reduction of TPyPz center at -0.75 V (blue line), second reduction of TPyPz at -1.23 V (green line) and bpy reduction at -1,5 V.

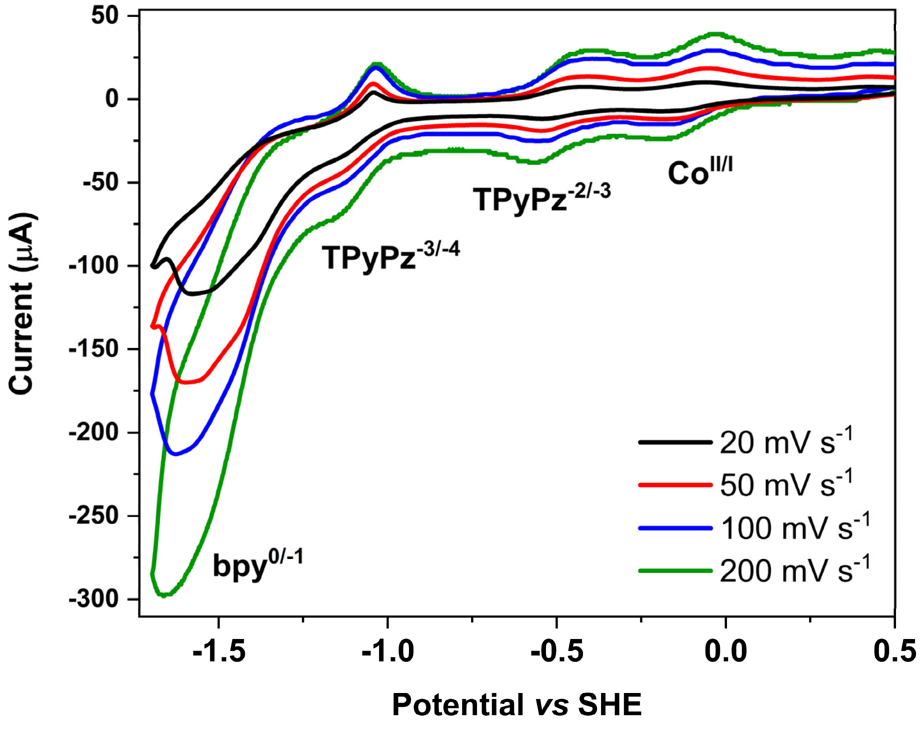

Figure S5. Cyclic voltammetry of TRuCoTPyPz in DMF containing $TEAClO_4$ 0.1 M at several scan rates.

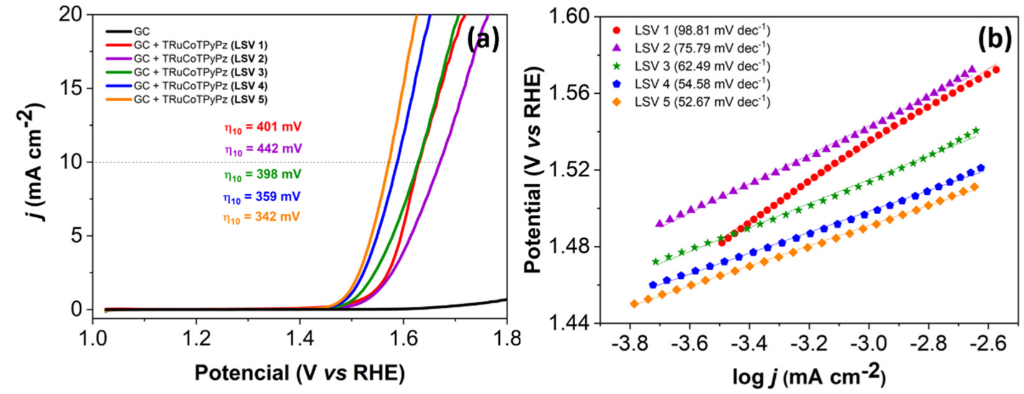

Figure S6. Successive LSV polarization curves at v = 5 mV s^{-1}, in 1.0 mol L^{-1} KOH solution (A) and corresponding (same color) Tafel plots (B) for TRuCoTPyPz. The working electrode was constantly rotated at 1200 rpm to remove the O_2 bubbles (For interpretation of the references to color in this figure legend, the reader is referred to the Web version of this article).

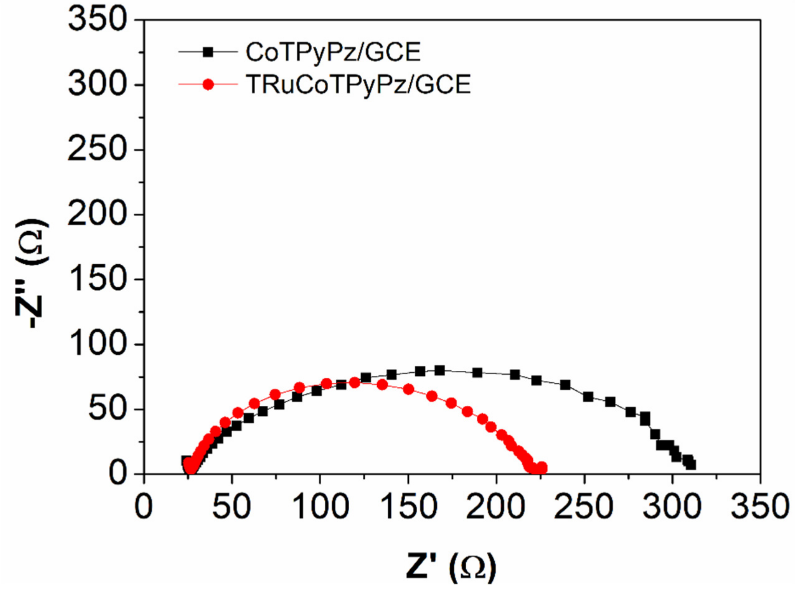

Figure S7. EIS data for GC electrodes modified with CoTPyPz and TRuCoTPyPz. Nyquist plot at DC potential of 1.573 V vs. RHE. The EIS measurements were recorded superimposing an AC perturbation (ΔE = 10 mV) in the 0.01 to 10.000 Hz range, after 5 successive LSVs in the 1.025–1.625 V vs. RHE range, at a scan rate of 5 mV s^{-1}.

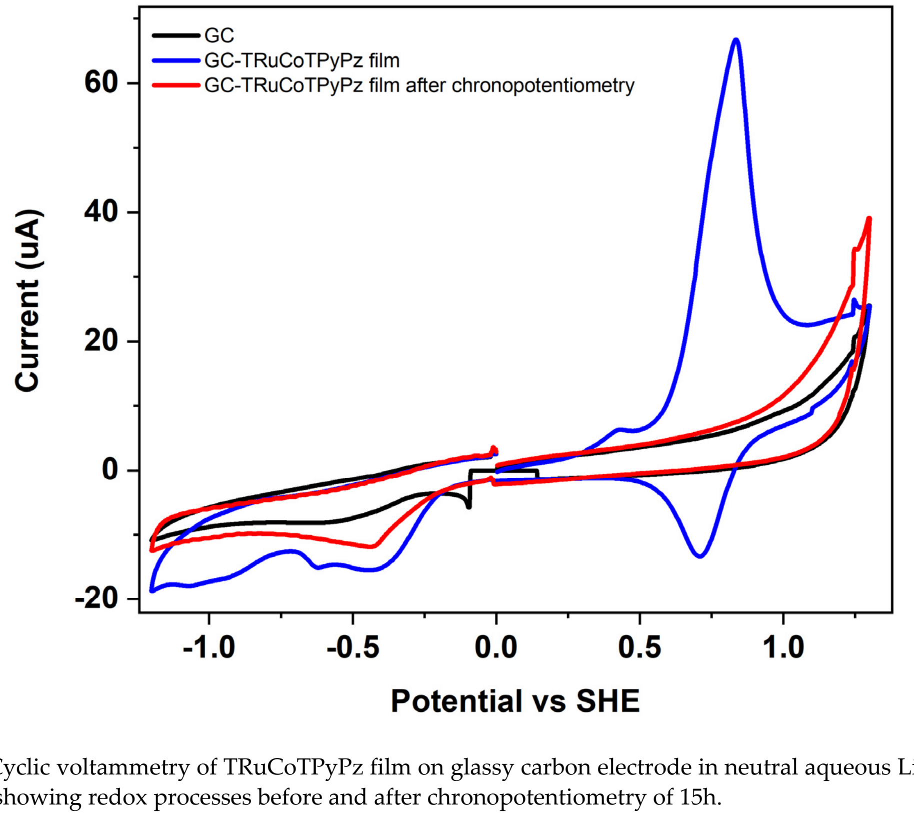

Figure S8. Cyclic voltammetry of TRuCoTPyPz film on glassy carbon electrode in neutral aqueous LiTFMS 0.1M at 50mV s^{-1} showing redox processes before and after chronopotentiometry of 15h.

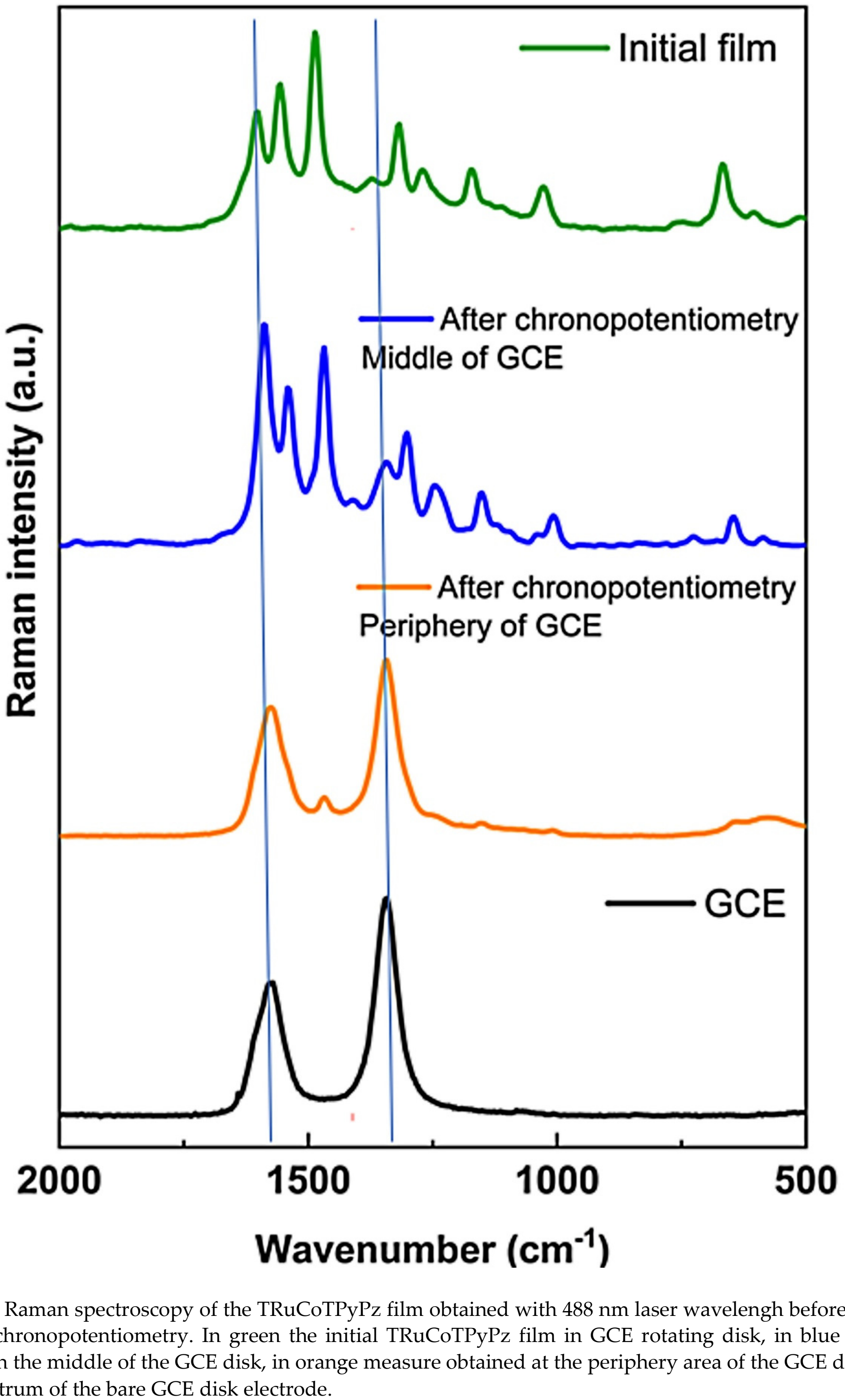

Figure S9. Raman spectroscopy of the TRuCoTPyPz film obtained with 488 nm laser wavelengh before and after 15 hours chronopotentiometry. In green the initial TRuCoTPyPz film in GCE rotating disk, in blue spectrum obtained in the middle of the GCE disk, in orange measure obtained at the periphery area of the GCE disk and in black spectrum of the bare GCE disk electrode.
